# Innovative Self-Confidence Webinar Intervention for Depression in the Workplace: A Focus Group Study and Systematic Development

**DOI:** 10.3390/bs10120193

**Published:** 2020-12-16

**Authors:** Wan Mohd Azam Wan Mohd Yunus, Peter Musiat, June S. L. Brown

**Affiliations:** 1 Research Centre for Child Psychiatry, University of Turku, Lemminkäisenkatu 3/Teutori 3. floor, 20014 Turku, Finland; 2 Department of Psychology, School of Human Resource Development and Psychology, Faculty of Social Sciences and Humanities, Universiti Teknologi Malaysia, Skudai 81310, Malaysia; 3 Department of Psychology, Institute of Psychiatry, Psychology and Neuroscience (IoPPN), King’s College London, London SE5 8AF, UK; june.brown@kcl.ac.uk; 4 Department of Psychological Medicine, Institute of Psychiatry, Psychology and Neuroscience (IoPPN), King’s College London, London SE5 8AF, UK; peter.musiat@kcl.ac.uk

**Keywords:** webinar, web-based, self-confidence, internet intervention, depression, workplace, iCBT, blended CBT

## Abstract

Brief face-to-face self-confidence workshops were effective in reducing depression among the public. Technological advances have enabled traditional face-to-face interventions to be adapted using unique technology-mediated platforms. This article details the formative development of a self-confidence web-based seminar (webinar) intervention for workplace depression. The first section discusses a qualitative study that explores the feasibility and acceptability of adapting the self-confidence workshops into a webinar platform on employees in the workplace. The second section describes the systematic development of this new webinar intervention informed by the qualitative study findings, a published systematic review, and previous face-to-face self-confidence workshops. The qualitative study involves three focus groups (*n* = 10) conducted in a small organization. Three themes were identified relevant to the running of the new self-confidence webinars in the workplace: personal (content, time and duration preference, features of the webinar, individual participation, personalization), interpersonal (stigma from others, engagement with participants/presenter, moderated interaction), and organizational (endorsement from management, work demand). For the intervention development, the format, structure, features, and content of the self-confidence webinar intervention are described. Features such as file sharing, virtual whiteboard, live chat, and poll are explained with the intervention primarily based on cognitive behavior therapy and coping flexibility concepts.

## 1. Introduction

The original large-scale psychoeducational workshop series by Brown was introduced in 1999 and set up in Birmingham as part of the WHO “Healthy Cities” campaign. This city-wide campaign provided health promotion programs including physical exercise, relaxation, reducing accidents, and stress management to the public. The psychoeducational workshop was conducted for free on Sundays in a leisure center. It aimed to provide accessible intervention for anxiety to the public. The results showed that the workshops were effective and reached many of those who did not previously consult their general practitioner (GP) [1].

However, when a similar strategy was implemented to conduct psychoeducational workshops that target depression, the results were less promising. It was reported that a relatively low number of people were recruited, and most had already been referred to specialist services [2]. Due to the close relationship between self-esteem and depression [3], it was then decided to change the title of the workshop from depression to “self-confidence” for the next workshop [4]. The results were very encouraging. Apart from showing improvements in depression and self-esteem when compared to the controls, nearly half (40%) of the participants had not consulted their GP. This highlighted the success of using the more non-diagnostic label title for the workshops in reaching those reluctant to seek help for depression. Notably, a result from a naturalistic follow-up showed that the effects of the workshop were also maintained at a two-year follow-up [5]. 

Similarly, the self-confidence workshops have also been shown to be clinically effective in reducing depression in a large open multicenter randomized controlled trial (RCT) study with an effect size of 0.55 when compared to the controls [6]. Furthermore, the study also reported that the workshop managed to reach 25% of participants who had not consulted their GPs and 32% of participants were from black and minority ethnic groups. Despite these very encouraging findings, the previous workshops were shown to be unable to attract those who are employed [6,7]. One possible explanation is that these workshops were all developed for a traditional face-to-face group format, which is not easily accessible to those at work, possibly because they may not have seen the publicity in GP surgeries, libraries, and other community centers. 

This article describes the systematic development of the self-confidence webinar intervention for depression in the workplace. The United Kingdom’s Medical Research Council (MRC) framework emphasizes various key elements related to the development of complex intervention before embarking on the evaluation phase. This framework proposes that the development of an intervention needs to be followed by a phase focused on feasibility and piloting [8,9]. The development phases also require a systematic accumulation of superior evidence preferably through a systematic review [9]. Based on these recommendations, a systematic review on available workplace intervention for depression was conducted and published [10]. In accordance with the emphasis on process evaluation observed in the updated MRC framework [11], a qualitative study was conducted that aimed to explore the acceptability, feasibility, and potential barriers to self-confidence webinars in the workplace by obtaining views of members of the potential target group of the intervention, that is, workplace employees. To capture a wide range of employees’ thoughts on such interventions, a qualitative methodology, focus groups, was chosen. These findings were then integrated with the materials from the latest self-confidence workshop for depression by Brown and colleagues [6]. Therefore, the aim of this article was to develop a self-confidence webinar intervention for depression that is suitable for delivery in the workplace and informed by the systematic review [10], proven face-to-face program by Brown and colleagues [6], and the qualitative empirical study.

## 2. Materials and Methods

This article describes the development process of an innovative self-confidence webinar intervention for depression that is suitable for delivery in the workplace and informed by a systematic review [10], proven face-to-face program by Brown and colleagues [6], and a focus group study. 

The focus groups were used to explore the acceptability and feasibility of self-confidence webinars for depression in the workplace. A sample of voluntary employees from an organization was recruited for the study. A total of 10 employees agreed to take part in the focus group. Participants were recruited from a small private organization specializing in media relations, consumer marketing, and reputation management. The focus group sessions were conducted at the premises of the organization, located in London. The focus group study was granted ethical approval by the King’s College London Research Ethics Committee (PNM 13/14-156). Having previously expressed interest in the study, the first author contacted the gatekeeper of the organization and provided detailed information on the study. The gatekeeper then approached the participants and provided them with information about the study. The focus group sessions were conducted in an environment familiar to the participants in a meeting room regularly used by the group. Prior to the focus groups, participants completed the written consent form and a brief demographic questionnaire. Each focus group lasted between 55 to 60 min. The discussion was steered by a topic guide as outlined in Table 1. Participants were encouraged to make comments without having to reach a consensus. The audio of the sessions was recorded with their consent and were later transcribed verbatim using NVivo and Transcribe Wreally, from which any personal identification information was removed. Data from the focus group was analyzed based on the thematic analysis approach in identifying, analyzing, and reporting patterns of themes within data [12].

## 3. Results

A total of 10 employees voluntarily agreed to take part in the focus group. Three focus groups were conducted, with three or four participants in each group. Table 2 shows the demographics of the sample.

Practicalities and barriers to conducting self-confidence webinars in the workplace were identified. These could be grouped into three themes relating to personal, interpersonal, and organizational-level factors. For each theme, there were several subthemes, which are summarized in Table 3 and discussed in the next section. 

### 3.1. Theme 1: Practicality and Barriers on a Personal Level

#### 3.1.1. Subtheme: Content

The employees generally agreed that the relevance of the content and aims of the intervention would influence their motivation to participate in a particular webinar.

“Content if it’s relevant to you or your job, or your life at some point then, you know it could be however long you want because people will always engage with the topic.” (R2)

“So I think I want to have a really clear in my mind the structure and what actually they are going to talk about, what I learn before I would sign up to that, but I would not rule it out completely if it is not something really I wanted to do.” (R5)

Furthermore, most had attended webinars that were more work-related, such as using software or attending training about more work-related issues.

“It’s about learning to develop some work-related skills; it was a training tool rather than a discussion tool.” (R9)

“So, the really good ones, particularly I mean most I have done, have been around software, to practically going through a tool online rather than they come to the office and show it to us that takes too long.” (R2)

However, almost all agreed that a webinar that focused more on the well-being of the employees might interest them.

“Actually I would do a lot on well-being, workplace well-being just because that’s what I’m interested in, and that can be implemented in the office.” (R4)

“Something that is useful to me and my personal life and maybe I have an issue with. So I think I would probably would be more motivated to do that more so hmm than work.” (R6)

#### 3.1.2. Subtheme: Time and Duration Preference

The employees reported different preferences with regards to the suitable time (e.g., early before starting work, just before lunch, up to 3pm) and duration of the proposed webinar. Nonetheless, most agreed that due to its work-related nature, it should be done during working hours rather than on the weekend.

“Yeah they schedule kind of like pre-lunch time at 11, so you can have a good time to get all your first priorities out of the way. Then you can join the webinar and in that way it’s quite useful.” (R6)

“I’ve got a view that early afternoon until 3 o’clock is quite a good time in terms of you have had your lunch, you have come back to your desk, and sort of you might have some time after that. You need to carry on with work, so it should not be the first thing of the day and not at the end of the day obviously.” (R5)

“Morning, well I think I definitely would give it all of my time in the morning, when I have got the whole day ahead of me and then, you know when it comes to 3, you obviously have a bit more crunch at that time.” (R10)

It was also agreed that a webinar is different than a traditional seminar because the participant will be in front of a computer screen rather than having a face-to-face view of the presenter and other participants. Therefore, all employees suggested that webinar sessions be no longer than one hour.

“It can be quite hard to keep engaged and keep focused on something if it’s more than 30 or 45 min. I reckon two hours will be quite a big ask especially when you spend a day like staring at the screen.” (R6)

“I always feel like half an hour and 15 min of questions and answers is good. So if you were really busy, you can just watch the first half hour, because not everyone wants to ask questions, yeah.” (R1)

“Another thing, the length would make a difference to me, if it was anything 45 min to an hour max, I just don’t think I can pay attention.” (R9)

Rather than having a session with a longer duration, the employees thought that having breaks or multiple sessions would be much more suitable than having the whole session at once.

“It is based on how interesting the last one is, then I will turn to the next one kind of thing.” (R4)

“I think would be better to have it 30 min in a number of days, one day a week rather than having the whole two-hour session in one day, so you can set aside some time for each day in a week.” (R5)

This was because the employees have their daily work tasks to do and it would be inappropriate for them to delay those tasks for too long in a day.

“Yeah there’s another thing about the thought that you can’t just disappear for half an hour, for every reason you need to be at your place somehow.” (R7)

“I think it depends because the ones I did were work-related, so in terms of people around me it’s actually fine for me to do it at my desk during working hours. But something like well-being training might not be received the same way by my colleagues, where I will be doing something that’s not directly related to work, so I think maybe that could be something that needs to be considered.” (R5)

#### 3.1.3. Subtheme: Features of Webinar

The employees also suggested that the design of the webinar and its features would be important. Generally, the webinar needs to be interesting by combining various aspects of visual and interactive features such as pictures and videos.

“I think if you want to have a screen there must be something there, if it’s just a voice coming out, I think I would move away. Even if it’s a video of someone talking, there has to be something or if it’s like a 30-s animation or something like that, that would keep your attention.” (R5)

“Yeah I think diverse range of visual content and interactive content and that will do, so yeah.” (R1)

“People you know might be more interested with the nice spread designs, with nice soothing colors. I don’t know, sometimes you look at a website or attend a webinar and literally the layout designs make you like it.” (R4)

“If it’s a mix with some visual stuff, a group conversation and yeah some kind of interactive talking on the headset or typing stuff in mm I think that’s more likely to keep people’s attention. Yeah.” (R8)

While the typical webinar is conducted live and most of the discussions are conducted during that time, employees felt that other resources or brief homework may also be helpful for their learning experience.

“If you would provide some links to potential relevant information such as about mindfulness, that would be one aspect people would refer to or if there’s a work sheet attached that is about something. So they don’t have to do it but compliments what they talked about. Or maybe a link to an app that does the same thing, or reminder for mindfulness for next week or something.” (R4)

“I would like it to be hmm…some resources where I can reflect back. So that would be good, or emails.” (R7)

“And that made me remind of what was in the session that you’ve just done, because when you are engaged in the webinar, it comes to a point when you can’t remember. So if you could find some of the things that have been talked about, after the session, maybe that would be good.” (R6)

“Yeah there might be something or resources that were in the background or something that you don’t have the chance to write down, so it might just be useful to have that captured somewhere.” (R5)

Another important aspect of the webinar for the employees was the technical ease of using the webinar itself.

“Yeah I agree sometimes when you have technical problems in linking up, it can just put you off the whole idea. Or if it’s too complicated to join and if it seems too much of a hassle.” (R6)

“Sometimes you have to add, download a little program to run in your desktop and I think, with something like this it would be better to have it in the browser so that if you are working with laptops that isn’t yours or somewhere within the workplace you don’t have to bring it on to the system, yeah.” (R6)

“So just a kind of thing where you just need to go to a web page and log in with some pre-login details, nothing more complicated than that.” (R8)

Additionally, as conducting the webinar requires running a technical program, an employee also raised the need to have technical support available during and before the webinar session.

“Yes that’s true actually, yeah, once you have started you need some kind of support system in case some people are struggling.” (R9)

When asked about the need for a recorded version of the webinar, most employees thought that there would be more benefits in a live session of the webinar than a recorded one.

“Yeah my attention span on the pre-recorded ones was not very good because I knew that was no danger of me missing something. It was pre-recorded so I can just skip up or I can watch the whole thing again. I think ultimately I was paying less attention to it rather than the live one. And I would have felt less bad about kicking out completely with that to be honest.” (R6)

“I think obviously the nature of the discussion is that people are just more naturally engaging when in live, or when you’re presenting something, very straight to the point whereas when you have live discussion, it can be more engaging, and encourage people to get involve with the conversation as well rather than just listening to for half an hour completely.” (R5)

“Hmm we probably learn less with the recorded format, but if you were really busy and can’t get anywhere so you can watch it in your own time, yeah.” (R8)

#### 3.1.4. Subtheme: Level of Participation

It was also suggested that some participants might be more comfortable than others in taking part, while others may be content with reading and looking at other people’s responses.

“I think the thing I just said, I mean where people feel like they might be forced to participate, that might put people off so I think it’s good to have the option but to make it clear that you don’t have to engage if you don’t want to, you could just listen.” (R9)

Furthermore, the level of participation may also relate to the possible communication mediums of talking or typing.

“Something interactive, like talking on the headset or typing stuff, hmm I think that would be more likely to keep people’s attention.” (R8)

#### 3.1.5. Subtheme: Personalization

A self-confidence webinar was viewed as quite personal for the employees, as it relates to the individual rather than to work. Hence, some thought that a personalized element of the program was also important.

“I think quite personalized maybe in a way, which you don’t always get with webinars because they sort of aim it at delivering information to save amount of time, I don’t know but maybe something a bit more about you rather than just things in general, I suppose.” (R6)

“Yeah I think personalized is important if someone to be confident about themselves and also I would believe, it’s not just giving me some general stuff, I think it has to be completely tailored to why and how a person feels, that would be better.” (R7)

Some also suggested that prior screening/assessment on baseline scores or what the problems are prior to the webinar session may also help to make the session more personalized to them.

“I’m going to say something like testing or sort of questionnaire you can fill up beforehand, I know it’s quite difficult because so many people are involved in a session but you need to sort of have an understanding of what the person’s problems are, and probably want to find out before you can really engage with them, I think.” (R5)

### 3.2. Theme 2: Practicality and Barriers on an Interpersonal Level

#### 3.2.1. Subtheme: Stigma from Others

Stigma was also a concern for most of the employees, especially among their colleagues, particularly when doing something personal in the workplace related to mental health.

“Hmm especially if it was, hmm, yeah something like mental health, you either want to be able to type or being in another room with different environment, not general office environment. Hmm, yeah.” (R3)

“Also the culture in terms of how people accept things, hmm, well-being and stuff, things as quite important, it’s like thinking being able to do it but also nothing judged when doing it.” (R6)

“Like if it turns out the guy next to you was doing it, you would have no way of not identifying him.” (R4)

Besides that, if the webinar were to be conducted during working hours, some also raised concerns about justifying their decision to participate in the webinar as it would be something indirectly related to work.

“I think it depends ’cause the one I did were work-related, so in terms of people around me it’s actually fine for me to do it at my desk during working hours, but something like your suggestion or the other examples it might not be received the same way by my colleagues, where I will be doing something that’s not directly related to work, so I think maybe that could be something that needs to be considered.” (R5)

#### 3.2.2. Subtheme: Level of Engagement with Presenter/Participants

Another important element that emerged is the interaction between the participants of the webinar. While some would be comfortable interacting with others, some would only be interested in gaining more information passively, from other people’s responses, rather than directly interacting with the other participants.

“I think I do want to know who they were because, for example there will be about 10 people who will be listening to the same thing. You want to at least to know a little bit about them, I want to at least know roughly who they were, hmm, even if we have, I don’t know, nicknames or something. It has to have a sense of sort of personal link with them I think.” (R5)

“I’m interested to see what they got to say, but I don’t want to get into a discussion with them directly.” (R9)

For some, the topic of discussion would also contribute to their openness with the other participants. They also felt that the suitable number of participants may also depend on the purpose of the webinar session itself.

“Honestly I think, again, it comes out to content, what topic it is. Because if it’s just to tell you some information then doesn’t really matter how many people around, but if the purpose of it is to interact with it, I think a smaller number may be better because particularly we are talking about personal topic sort of thing, you don’t want to broadcast that (laugh).” (R2)

“It all depends on the program but I would say not more than 20.” (R5)

Some employees also discussed the interaction between the participants and the webinar presenter. The webinar presenter was considered to be an important figure during the session and participants suggested that the presenter needs to be qualified, have skills to conduct the webinar, and at the same time continuously engage with the participants.

“Although before signing up I might be more interested to know who they are. So actually, the point of signing up, if it says a bit about who the presenter is, why they are qualified and why they might be interesting, hmmm, that might make a difference signing up in the first place.” (R9)

“I mean, not to have a mental health doctor for the next six weeks, that’s being a bit extreme, yeah, but just being aware that they have background in this field and they are knowledgeable about it, is enough for me.” (R5)

“The worst thing is when we have someone who is, obviously that’s not their fault, but is a bit nervous, and kept saying ‘that was really interesting, thank you’, I’ve already heard that 40 times so I guess just being concise and very eloquent and informative.” (R10)

#### 3.2.3. Subtheme: Moderated Interaction

Due to the nature of the webinar program, all interaction is displayed via a screen of a computer or an electronic device. Therefore, the employees suggested a need for moderated interaction whereby responses can be filtered and organized accordingly in order to maintain the appropriate flow of the program.

“So you can just type in a question and then the moderator of the webinar will receive the questions in like a big feed, and then at some point during or after the presentation they will answer those questions. So you can kind of interact with other people but you are not interrupting the session, which is really good.” (R2)

“Basically I don’t want a person just reading it, but I want that they have control over our interactions, not just one person consistently talking.” (R10)

“So I want to see if someone else is asking a question and what the responses are. I guess if you got something to say, you can say, but it’s all being filtered to the presenter and they decide whether your opinion is valid. Well because otherwise, I don’t know I mean, but you could have people being rude or inappropriate.” (R9)

### 3.3. Theme 3: Practicality and Barriers on an Organizational Level

#### 3.3.1. Subtheme: Endorsement from Management

The method of approaching the organization in the first place also came up. Some employees suggested that endorsement by higher management would be important for them before they would decide whether to participate in the webinar.

“If it was something been mentioned by line management, to say that, you know, ‘this is a really good system, why not try it’, etcetera, and they will help you sort of, not manage your diary, but just you know, for them to be aware where are you going while attending the webinar, so it’s not that you just disappear for half an hour.” (R1)

“Yeah I think if it came from the general management, about this well-being thing, or if it came from the team leaders, ‘I welcome you to do this’, I would do it.” (R7)

“Yeah I think that would make a difference to me if someone said, or my boss said, she said, ‘Yes I’ve done this and I found it positive’, it would make me more likely to take it up.” (R9)

Some employees thought that they would prefer their participation to be confidential instead of known across the organization and by their managers or supervisors, although they agreed that permission from the organization for the employees to take part is important.

“And yeah, I think if any line managers specifically talk and say they have done it, that would put me on. And also knowing your participation is not going to be logged in some way.” (R9)

“And I think, I think it would be better to hmm…work on the assumption that people who are going to attend somehow got the OK from their boss, rather than checking that it’s alright. I mean I wouldn’t sign up to a webinar without telling at least that I would be doing that at some point during my week. So hmm, yeah, I’ll check if I sign up to something and then rather than getting the message ‘A has sign up for this webinar on Thursday morning…’, it would be weird. I don’t think the management will find this useful.” (R8)

#### 3.3.2. Subtheme: Work Demands

When asked about other aspects that may hinder their participation, employees stated that the work demands were an important factor that could influence their motivation to participate in the webinar.

“The level of work, I think that’s the main thing. Yeah I think when you listen to the webinar, without doing your job, yeah I just think however good people’s intentions are to do it, it’s a lot for people to get involved with.” (R1)

“Work. If your boss or your team wants you to do something important, so, that would definitely put me off from doing it I think.” (R6)

## 4. Discussion

### 4.1. Principal Findings

The findings clearly indicate that a self-confidence webinar is likely to be acceptable to employees as potential users and that it can be feasibly developed. The qualitative study elicits useful information to inform the development of self-confidence webinars in the workplace. 

The results can be categorized into three main themes: personal, interpersonal, and organizational levels. Generally, the personal aspect is the most discussed category compared to the interpersonal and organizational levels. This suggests that the individual acceptance of the intervention is the most important aspect in order to ensure that the intervention be accessed by the employees. Of this, it is important to understand what may influence individuals to take part in a workplace health program that fits their needs and enables them to gain greater control over their health [13]. Besides, it is important to highlight personal factors in the development process as a significant body of research has provided evidence of the effects of individual attitudes or attitudinal barriers towards mental health help-seeking [14]. 

Within the context of this study, there are several subthemes that emerged concerning personal factors. Consistent with a previous qualitative study on why people opt to use internet intervention [15], it was found that the content of the intervention may influence the motivation of the employees to take part in the webinar. This is in line with the social psychology perspective wherein individuals are more likely to process messages that they perceive as meaningful to them personally [16]. Based on previous experiences, some employees attended webinar sessions that were more technical-based and directly related to their job. Although a self-confidence webinar is not directly related to their job, most did think that well-being in the workplace, rather than just skill-based workshops, may also be relevant and important for them.

Although the employees had different preferences about the best time to conduct the webinar, they were in agreement that a session needs to be short, concise, and divided into chunks. This is due to the nature of the webinar itself, in which participants need to pay attention to the computer or device screen continuously when undertaking the program. It is acceptable to attend a face-to-face seminar for half or one whole day, but it would be less practical to do the same with webinars. Furthermore, given the work-related nature of the webinar, most employees were in agreement that the webinar should be conducted during working hours. Another related factor is that full-time employees spend an average of 37.4 paid hours per week at work [17], making the limited available time outside work reserved for other activities. 

The focus groups also highlighted the importance of having an easily accessible webinar with the right features that can cater to the needs and technical capabilities of the employees. One review suggested that due to time constraints, employees prefer programs and services that are easily accessible that come to them as much as possible, rather than the other way around [18]. It was highlighted that interactive and appealing interventions may contribute to higher participant engagement, better retention, and may influence intervention effectiveness [19]. People often associate technology with visual and interactive features, but it is also important to consider the technical ease of using a specific program. While it is common for people to want interesting visual and interactive features, it needs to be balanced with the ease of use and the technical specifications required to accommodate these features.

Furthermore, the self-confidence webinar is intended to allow people to actively interact remotely while being anonymous. Despite the anonymity, participants may decide to take part actively or passively. The focus groups suggested that some people may be content to just see what others have to say, rather than actively contributing to the session. Despite this passivity that may appear to contradict with the self-help elements of the self-confidence webinar, a meta-analysis reported that passive psychoeducation programs have also been shown to reduce symptoms of depression and general distress [20]. Furthermore, it could be that the employees mistakenly perceived webinars as more oriented towards passive involvement. This may be attributed to the past experiences of the employees in attending skill-based webinars during which they primarily only received information from the presenter. While participants should not be forced to engage in the sessions, a more creative webinar setup that covers more personal aspects may be likely to increase participants’ motivation to actively participate in the session. Even if they do not, some benefits can still be retained regardless of the active or passive involvement of the participants.

The use of a more personalized approach is important when designing successful workplace health promotion programs [21]. Furthermore, a tailored internet-based intervention was also more effective when compared to a standardized internet-based intervention for more severe depression [22]. Self-confidence is a personal topic and the focus groups suggested that the element of personalization is important for the webinar. Although it is difficult to tailor the whole program to every individual’s needs, the employees suggested that prior assessment related to the webinar content could help the intervention be more personalized. The fact that a webinar requires the role of the presenter to run the program may also allow identification of employees’ needs and discussion about what the webinar may provide during the initial session. Using the available features from the webinar, employees may also ask questions directly to the presenter or other participants throughout any session to acquire information that fulfils their individual needs.

Attending the webinar would require the participants to stop their current work task for a while in order to pay attention to the webinar program. The stigma of mental health problems is common in the workplace [23,24,25], and employees from the focus groups also argued that the topic of the webinar may create stigma among their colleagues, as well as the organization. However, making the webinar available to all employees and obtaining permission and endorsement from the organization were likely to reduce this stigmatization. Webinar sessions are pre-scheduled and can be treated like other types of training that are commonly conducted in organizations during standard working hours. Besides that, the webinar also aimed to reduce depression by targeting self-esteem and used the non-diagnostic label of “self-confidence” for the webinar program, as it has been shown that that non-diagnostic label may increase depression intervention take-up [4,6].

The focus groups also discussed two types of engagement during the webinar: between the participants, and with the presenter. Although not all employees were interested in interacting with other participants, the majority agreed that even if they did not, they could still benefit by looking at the responses or suggestions provided by other participants. This echoed with the previous point on the personal level of participation where employees may decide to take part actively or passively. The group nature of the webinar and its platform in using the internet mirrors that of an online discussion forum that provided benefits for individuals with stigmatized conditions such as depression in terms of improving and managing depressive symptoms, especially when members identify with other members [26]. For the webinar, although identification between group members was limited to their names and nicknames, participants could still benefit from gathering valuable advice and sharing experiences anonymously [27]. This process may then eventually allow the participant to develop a sense of closeness with other virtual group members experiencing the same issues or problems [28,29]. 

While the level of engagement between participants and presenter depends on the individual concerned, the employees agreed that the session should be moderated systematically. This is because the interactions are mainly conducted through the screen via audio or visual stimuli. Therefore, the presence of a moderator capable of filtering and organizing the responses from the participants was deemed to be useful to maintain the flow of the webinar. Likewise, a review of health-related online support groups emphasized the importance of moderated interaction to make sure that the discussion is consistent with the purpose of the group and reduces potential disruption caused by the anonymous members [30]. Significantly, unmoderated and unstructured internet-based support platforms have been shown to be detrimental, in which individuals with psychiatric disabilities who participated reported higher levels of distress over time [31].

From an organizational perspective, most employees agreed that they should attend the session while at work, resonating with the suggestion that the session should be conducted during working hours. Therefore, permission from the organization is important. The employees agreed that they might be more interested in taking part if the webinar was endorsed by higher management. While this may automatically mean that the organization allowed its employees to take part in the webinar, encouragement from managers for their employees to participate would be even more encouraging. This is in line with previous studies that suggest senior management commitment, and supervisor support and leadership, are crucial in the recruitment and participation of employees in workplace health promotion programs [21,32]. The importance of endorsement and support, however, must be consistent with the confidentiality aspect. The employees suggested that their participation in the webinar must be confidential across the organization. This is consistent with findings in a previous qualitative study that suggested confidentiality is one of the key aspects that prevented employees from seeking help for mental health [33]. That study was conducted in a healthcare setting, where confidentiality is crucial across the organization, which may suggest that attention to confidentiality may also be vital for organizations from different backgrounds, such as those for this current study.

Additionally, a qualitative study identified workload pressures and lack of time to access interventions as key factors that inhibit employees from seeking mental health support [33]. Within the context of this study, the employees still felt that their work was more important and would not hesitate to drop their interest in the webinar if any urgent, important work-related demand arose. As previously suggested, workload, workflow, and production levels may need to be considered in order to make sure that employees can take part in workplace health programs [32]. Despite the purpose of the webinar, which was oriented towards the workplace setting, it is important to consider the amount of time and the availability of employees to switch their attention from work duties to attending a webinar for their self-development.

### 4.2. Intervention Development Process

**Figure 1:**
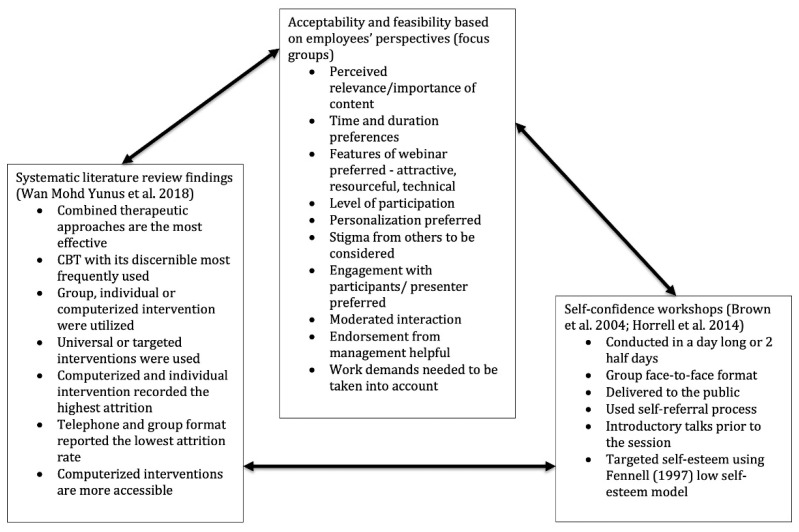
Integration of systematic literature review findings, focus group study, and existing self-confidence workshop.

The intervention development procedures are in line with the development and feasibility/piloting phases of the United Kingdom Medical Research Council (MRC) framework for the development and evaluation of complex interventions [1]. This section entails an integration of three components: the outcomes of the systematic review [33], the results from the focus groups, and the original self-confidence program [8,9]. Figure 1 displays the elements that were integrated in order to develop the self-confidence webinars in the workplace.

#### 4.2.1. Combined Therapeutic Approach

The webinar intervention integrates several components: evidence-based cognitive behavior therapy (CBT) self-confidence workshops [4,6] upon which the webinar program is based and coping flexibility [34]. In the literature review, successful coping may require more than simply the attainment of coping skills realized through CBT. CBT interventions were the most frequently used in the workplace and showed encouraging results. Additionally, the addition of the meta-skill of coping flexibility seems to be another notable feature. This feature elevates an individual’s sensitivity to the degree of controllability and the suitability of coping strategies that can be applied [34].

This intervention approach differs from that of the more procedural work-related webinars that are commonly conducted in a workplace setting. Those work-related webinars and trainings are usually only oriented towards technical skills. On the other hand, although well-being webinars may be oriented towards the workplace, the skills that can be learned through CBT and coping flexibility may also be used to other situations outside of work. Additionally, because the skills can be applied in other situations, this webinar intervention may have the potential to attract employee interest regardless of their working backgrounds. Although the webinar targets individual skills, this self-development process may also benefit the organization. There are arguments that well-being intervention programs conducted in the workplace can be advantageous to an organization because of reduced costs due to reduced absenteeism and medical expenses. A meta-analysis revealed that every dollar spent on a workplace wellness program led to a USD 3.27 reduction in medical costs and a USD 2.37 reduction in absenteeism costs [35].

#### 4.2.2. Duration and Number of Sessions

Issues pertaining to the duration of the intervention include the decision to compromise either the effectiveness of the intervention or the adherence of a technology-mediated intervention. A previous study suggested that a brief version of online CBT for depression was not as effective as the extended version [36]. On the other hand, it was reported in a systematic review that other than disorder-specific factors, the predictors of internet intervention adherence also include the treatment length [37]. A review also concluded that the weighted average dropout rate from internet interventions for psychological disorders stands at 31% [38]. Feedback received from the focus groups implied that employees favored brief and concise sessions over several sessions, preferably less than one hour per session. While they agreed that multiple self-confidence webinar sessions are practicable, these sessions would need to fit into working hours. This is an indication that the duration of the webinar needs to be short and the number of sessions in the webinar intervention also ought to be limited. 

Nevertheless, it is important to take into account the therapeutic approach of CBT, which requires coverage of certain content that is essential for it to be effective. It was felt that five one-hour sessions of CBT and one one-hour session for coping flexibility was the minimum possible. Although a reduction in the number of sessions may result in more participants remaining until the end, this move can have negative effects on the efficacy of the intervention. While this may seem a heavy commitment in terms of time, the interest of participants can be maintained through the development of an attractive and appealing intervention. This echoed the suggestion by Ludden and colleagues highlighting the importance of design research in the implementation of a web-based intervention and the necessity to improve adherence [39]. They suggested that in addition to effectiveness, it is also important to develop interventions that are desirable, compelling, and delightful in order to increase the acceptance of and adherence to these interventions.

#### 4.2.3. Universal or Targeted Intervention

The focus group discussion showed that the effects of stigmatization need to be taken into consideration. This brings into question the very nature of the intervention. Should it be generally available for the whole working population, or restricted to a select group of individuals? A review and meta-analysis revealed that universal workplace interventions exhibited small but significant contributions towards the reduction of depressive symptoms among employees [40]. They argued that while universal interventions are not expected to generate sizeable individual effect dimensions, they have the potential to positively affect the entire workforce. As universal interventions are directed at an entire population, they may approach individuals at different stages of readiness to seek help [41]. This is especially important among individuals who are hesitant about seeking treatment or disclosing symptoms due to fear of stigmatization, negative perception by managers, influence on employment, or revealing weaknesses to others [25,42,43,44]. With this in mind, the self-confidence webinar intervention was offered universally to all employees in the organization who thought they could benefit from it. Besides that, the decision to participate or otherwise was left entirely in the hands of each individual employee, making it an open-access intervention.

#### 4.2.4. Group or Individual Intervention?

In a comparison between individual and group stress management programs in a workplace setting, it was discovered that participants were more in favor of the former than the latter. Interestingly, there were hardly any indications that the level of effectiveness on depression outcome was any different between the group and individual stress management programs. While some employees in the focus groups thought that a personalized and individual intervention would be preferable, it is undeniable that group intervention is more realistic, viable, and cost effective in a workplace environment [45]. Previous studies have reported the cost effectiveness of group CBT as compared to individual CBT [46,47]. In a review of 36 studies related to cost effectiveness in the treatment of mental disorders, group CBT was superior when compared to individual CBT [46]. They also reported the benefits of group therapy, which included the method for treating a larger number of people, the opportunity for group members to perform as co-therapists, the availability of group support, the opportunity to identify and share common experiences, and other advantages that come with being part of a group. Additionally, the systematic review also reported a lower attrition rate among those employees who attended group-based interventions as compared to individual-based interventions [10].

#### 4.2.5. The Importance of having Initial Face-to-Face Contact

It was interesting to note that the original self-confidence workshops were complemented by an introductory session before the commencement of the workshop. The main aim of this introductory talk was to “sell” the workshops to potential participants by making it reasonably positive and upbeat. From a potential participant’s perspective, this would allow the participant to decide whether the workshop would be helpful for them before deciding to take part. This is consistent with the findings from the focus groups in which employees preferred to have an understanding of the content of the webinar in advance before deciding to take part. Previous research also suggested that curiosity about the intervention as well as the content of the intervention were some of the important factors that could make people engage with an internet intervention [15]. 

Additionally, for this webinar the introductory session was considered important because it also allowed the potential participants the opportunity to meet the presenter and research team face to face as well as to clarify the procedure of attending the webinar, especially among those who were not familiar with its technical procedure. One critique of internet interventions was that they lack the physical connection between the individual and therapist and can influence the compliance rate towards the intervention [48]. Since the webinar sessions were conducted remotely, the introductory session allowed initial contact to occur face-to-face before shifting into a virtual environment.

#### 4.2.6. The Need for a Moderator

Face-to-face intervention usually utilizes synchronous communication directly between the therapist and individual. For instance, the workshops by Brown were conducted by two psychologists who delivered the content alternately [4,6]. Each one of them was able to manage any communication with the participants directly and face to face. Because the webinar provided a different platform (interface with a computer/mobile device screen) and was also conducted in groups, it was decided to have a moderator whose function was to manage and organize the flow of communication throughout all sessions. This was echoed by the findings from the focus groups in which employees were in agreement that the role of the moderator in each session was crucial. Evidence also shows that moderated interaction is crucial for online support groups to control the discussion between group members and minimize the risk of disruption from happening [30]. As the communication during webinars mirrors that of online support groups, it was decided that a moderator other than the presenter is needed in each webinar session.

### 4.3. Webinar Intervention Form and Structure

The webinar intervention for building up self-confidence in a workplace setting lasts for six weeks, with one session per week. Every session comprises an hour-long live webinar that includes homework as well as Q&A. To attend the sessions, participants can use a computer, laptop, or mobile device, including smartphones (iPhones or Android phones) and tablets (iPads and tablets). Thus, attendance at these sessions was possible wherever the internet was accessible. Prior to the webinar, consent from line managers or team leaders was obtained, as the sessions were run during working hours. Screenshots of the webinar intervention form and structure are available in the proof-of-concept study publication [49]. 

#### 4.3.1. PowerPoint Presentations

An innovative feature of this intervention was the use of a combination of a PowerPoint presentation with comics and animation videos. Text was not left out completely but restricted to a bare minimum so that participants did not feel overwhelmed by too much text during the sessions. Based on the utilization of the features mentioned above, the authors developed and adapted these files for the webinar intervention. Essentially, all sessions were presented on PowerPoint slides embedded with videos and images. 

#### 4.3.2. Comics

While comics are currently utilized mostly in the educational domain [50], they can also be successfully applied in the medical field. Notably, a survey found that the manga (Japanese comic) layout is extensively used in Japan for the communication of medical information, with the number of publications having increased since the first one in 1970, and particularly rapidly after 1980 [51]. Findings from the survey concluded that manga can be a powerful tool for transmitting medical information to the public, especially given that manga readers consist of all age groups. The application of comics in medical education and patient care has also been described as a creative and interesting way to educate about illnesses [52].

A study assessed an internet CBT (iCBT) with a manga storyline and showed that its utilization significantly reduced the level of depression in a workplace setting [53]. Admittedly, the effect size found was modest, but could be attributed to the universal nature of the intervention [40] and lack of therapeutic guidance [54] throughout the intervention. Moreover, a more significant effect was found in the results among those who had more severe depression at the baseline [53].

The comics for the intervention in this study were produced by a freelance artist. To ensure they were visually appealing, Adobe Photoshop was brought into play to color the hand drawings. Although the intervention was a universal one, the presence of the webinar presenter throughout the session enabled consistent and extensive guidance to aid participants’ understanding of every comic.

#### 4.3.3. Animation Videos

In order to increase the attractiveness and interactivity of this intervention, animation videos were included. This is in line with the cognitive load theory, which states that the employment of visual and auditory aids has the potential to extend the parameters of an individual’s memory capacity [55]. Compared to using text, content within a video can be more information rich, which is an advantage as it could allow for the possibility of tailoring the video to make it more interesting. Unsurprisingly perhaps, studies conducted within educational environments indicate that information supplied in the form of a video increased levels of attention and interactivity [56,57,58]. It has also been found that videos also pave the way towards greater emotional engagement compared to text, which could improve participants’ evaluation, judgement, and recall of information [59].

Each session of this webinar intervention included at least one animation video. Animation videos were utilized to (a) offer a comprehensible description of a concept or (b) exhibit a brief storyline or case study in a thought-provoking style. Discussions with the research team were held to determine the storyline, content, and dialogue for each video. Go Animate was employed for the production of these videos by the first author. This cloud-based online instrument enables the creation of concise video animation clips and also comes with a text-to-voice function. It facilitates the creation of animation videos from the basics, or from a wide range of themes, backgrounds, characters, and props [60].

#### 4.3.4. The “Interactive Zones”

This webinar allowed participants to respond immediately through the chat channel. This channel facilitated participants to type responses synchronously and immediately, and allowed responses from participants to be viewed throughout the session. Furthermore, the method of interaction through the chat panel allowed for more user control by the participants, whereby more careful construction of information could be made. This “delayed” nature of communication may provide participants with the opportunity to review, revise, or cancel their response before the information is sent to other people [61]. Previous studies have investigated the use of chat rooms with a chat function of a similar form. Chat rooms have been found to be effective for the reduction of depression in adolescents [62], for the promotion of HIV testing [63], and for the reduction of eating disorder symptoms [64,65]. The quickness of each response is what sets this method apart from asynchronous computer-mediated communications such as e-mail [63].

Notably, it must be remembered that these studies investigated chat rooms as the sole medium of intervention, whereas for this intervention the chat function was used as one of the many functions of the whole webinar. Likewise, chat rooms can offer the user an inimitable and interactive electronic communication avenue [63], two-way communication, peer support, and a source of information [66]. Given that other features were also utilized, this may provide an added advantage when using a webinar. However, it was asserted that chat rooms are open to exploitation as (a) the actual identity of the user is unknown and (b) a variety of communication threads can be present simultaneously [67]. In order to alleviate these risks, for this study the following steps were taken:

- Panels were made available mostly during the “Interactive Zones” slides, when the responses from participants regarding a specific topic assigned by the webinar presenter were solicited.
- The responses by all participants were filtered and managed by the moderator and only credible responses were exhibited on the whiteboard.
- Participants were constantly prompted to maintain their concentration on the discussion theme.

#### 4.3.5. Webcam

The webcam was an additional feature of the webinar that allowed participants to observe the presenter throughout the session. One study compared three modes of psychotherapy: face to face, real-time video conference, and two-way audio (analogous to telephone), and found that the disparity in process and outcome measures with regard to CBT delivered across these three modes was negligible [68]. That study would suggest that a webcam can increase the involvement of participants. This suggests that enabling the webcam of the presenter may increase the social presence and improve the efficiency of the session.

#### 4.3.6. Homework and Post-Session Enquiries

Each session concluded with the assignment of weekly homework. Though not compulsory, participants were encouraged to complete their homework for improved comprehension. Post-session requests for information from the presenter through the first author were available by e-mail. Queries were administered and accumulated by the first author and were addressed during the five opening minutes of the following session. 

### 4.4. Intervention Content

#### 4.4.1. Introductory Meeting

Prior to the commencement of the webinar, an introductory meeting for each participating organization was conducted. This introductory meeting had two main aims: (1) to explain the overview of the program and the technical procedure of attending the webinar (i.e., before, during, and after the webinar) and (2) to provide the opportunity for the potential participants to ask questions before deciding to take part.

#### 4.4.2. Theoretical Basis of the Webinar Intervention

This intervention merged the cognitive-behavioral procedures component adapted from the work by Brown and colleagues [6], which was originally derived from Fennell’s book, *Overcoming Low Self-Esteem* [69]. Fennell’s work was grounded in the cognitive behavior therapy established by Aaron Beck [3,70]. 

The cognitive behavior therapy (CBT) program by Brown and colleagues was evaluated in a large randomized controlled trial (RCT) on self-confidence workshop programs that were found to be clinically effective for the public [6]. However, not many employed people attended these workshops. The webinar therefore focused on employees and directed its attention more towards workplace concerns that embrace work-related undertakings as well as the communication among employees, subordinates, and colleagues. In addition, the final session touched on coping flexibility [71], which has been shown to be an important meta-skill with CBT in an RCT evaluation of interventions for depression in the workplace [34].

#### 4.4.3. Intervention Sessions Summary

**Figure 2:**
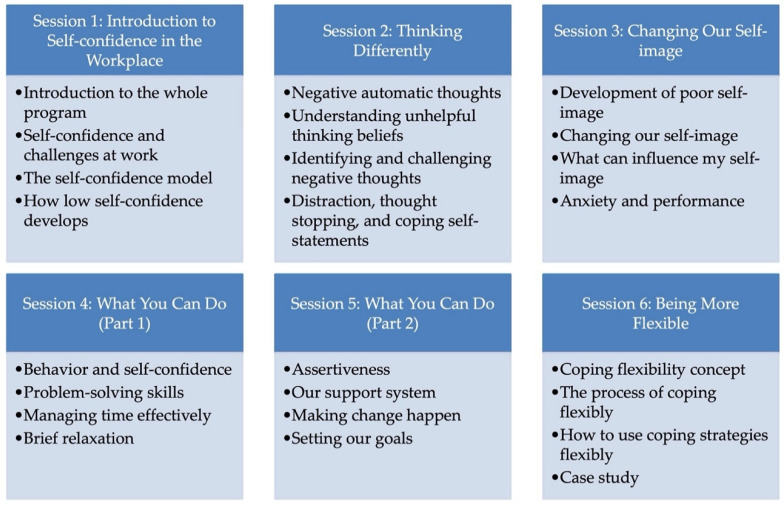
Summary of intervention sessions.

Following the introductory meeting, the intervention consisted of six sessions conducted weekly. The content of the sessions, largely based on cognitive behavior therapy [6] with coping flexibility [34], are described in detail in Figure 2.

### 4.5. Strengths and Limitations

The major strength of this study is the depth of data obtained from the qualitative focus group interviews that were conducted. The employees explored their ideas and discussed them with other participants. The richness of the data is valuable for the research, especially considering that this is a new intervention that has not been previously used. The findings regarding the practicalities and barriers of developing this intervention may not have emerged by using quantitative measures.

A weakness of this study is that the focus groups were only conducted in one organization, and only one male participant decided to take part. The participants were selected on a voluntary basis and the composition of the focus groups depended on the availability of the employees during the days of the scheduled focus groups. The focus groups were also conducted in a small organization, which reduced the number of potential participants that could have been recruited. Although the percentage of participants that took part was high, the results still only represented a small sample of 10 employees. It would be interesting to explore feedback from other employees from different organizations that may have different thoughts and suggestions regarding the webinar.

## 5. Conclusions

This study describes the formative development of an innovative self-confidence webinar intervention for depression in the workplace based on a qualitative study followed by the description of the intervention format, structure, and content. The focus group results indicate that the self-confidence webinar is feasible to be conducted in the workplace if due consideration is paid to personal (content, time and duration preference, webinar features, participation, and personalization), interpersonal (stigma, engagement level, and moderated interaction), and organizational (endorsement and work demands) aspects of the delivery of the webinar. Although some challenges may be present, employees seemed to think that the development of self-confidence webinar interventions for depression in the workplace is possible and promising. The next section explains the development of the self-confidence webinar intervention using Adobe Connect encompassing the intervention format, structure, and content. Then, the unique features of the webinar were used to present the content of the intervention. This involved the development of media such as a PowerPoint presentation, comics, and animation videos as well as other features available through the webinar. Additionally, the crucial roles of the “Interactive Zone” and chat panel were highlighted. The different roles of the presenter and moderator were also highlighted, and the content of each session and the theoretical foundations were outlined. This study provides evidence that a self-confidence intervention that was previously delivered through workshops can be adapted to a webinar format. Traditionally, interventions for depression in the workplace have been delivered face-to-face, whether individually or in a group. As with face-to-face interventions, the webinar intervention was developed according to evidence-based theoretical concepts. This intervention has also been evaluated in a proof-of-concept study [49].

## Figures and Tables

**Table 1:** Focus groups topic guide.

Topic Guide
1. Has anyone heard of webinars? Explain. 2. When do you think is the best time to conduct this webinar? Where? How long? 3. Are there any other important aspects that need to be considered to improve webinar acceptance in the workplace? 4. What could hinder employees from taking part in the webinar intervention? 5. How could this be improved? 6. Supposed you had one minute to explain what a good workplace intervention is. What would you say?

**Table 2:** Demographic characteristics of employees.

	Demographics	
Age (years old)	Range	23–32
	Median	26
Gender	Female	9 (90%)
	Male	1 (10%)
Ethnicity	English/Welsh/Scottish/Northern Irish	9 (90%)
	Others—Australian	1 (10%)

**Table 3:** Themes and subthemes.

Themes	Subthemes
Personal	Content
	Time and duration preference
	Features of webinar
	Individual participation
	Personalization
Interpersonal	Stigma from others
	Engagement with participants/presenter
	Moderated interaction
	Stigma from others
Organizational	Endorsement from management
	Work demand
